# Quantitative Real-Time PCR detection of TRPV1–4 gene expression in human leukocytes from healthy and hyposensitive subjects

**DOI:** 10.1186/1744-8069-4-51

**Published:** 2008-11-04

**Authors:** Giacomo Spinsanti, Raffaella Zannolli, Cristina Panti, Ilaria Ceccarelli, Letizia Marsili, Valeria Bachiocco, Francesco Frati, Anna Maria Aloisi

**Affiliations:** 1 Department of Evolutionary Biology, University of Siena, Via A. Moro 2, 53100 Siena, Italy; 2 Department of Physiology, Neuroscience and Applied Physiology Section, University of Siena, Via A. Moro 2, 53100 Siena, Italy; 3 Department of Pediatrics, University of Siena, Policlinico Le Scotte, Viale M. Bracci 14, 53100 Siena, Italy.; 4 Department of Environmental Sciences, University of Siena, Via P.A. Mattioli 4, 53100 Siena, Italy; 5 Anaesthesia and Intensive Care Unit, S. Orsola Hospital, Via A. Massarenti 9, 40100 Bologna, Italy

## Abstract

**Background:**

Besides functioning as chemosensors for a broad range of endogenous and synthetic ligands, transient receptor potential vanilloid (TRPV) 1–4 channels have also been related to capsaicin (TRPV1), pain, and thermal stimuli perception, and itching sensation (TRPV1–4). While the expression of the TRPV1–4 genes has been adequately proved in skin, sensory fibres and keratinocytes, less is known about TRPV3 and TRPV4 expression in human blood cells.

**Results:**

To study the gene expression of TRPV1–4 genes in human leukocytes, a quantitative Real-Time PCR (qRT-PCR) method, based on the calculation of their relative expression, has been developed and validated. The four commonly used house-keeping genes (HKGs), β-Actin (Act-B), glyceraldehyde-3P-dehydrogenase (GAPDH), hypoxanthine ribosyltransferase (HPRT1), and cyclophilin B (hCyPB), were tested for the stability of their expression in several human leukocyte samples, and used in the normalization procedure to determine the mRNA levels of the TRPV 1–4 genes in 30 healthy subjects. cDNAs belonging to all the TRPV1–4 genes were detected in leukocytes but the genes appear to be expressed at different levels. Our analysis did not show significant sex differences in TRPV1–4 cDNA levels in the 30 healthy subjects. The same qRT-PCR assay was used to compare TRPV1–4 expression between healthy controls and patients hyposensitive to capsaicin, pain and thermal stimuli: an almost doubled up-regulation of the TRPV1 gene was found in the pathological subjects.

**Conclusion:**

The qRT-PCR assay developed and tested in this study allowed us to determine the relative expression of TRPV1–4 genes in human leukocytes: TRPV3 is the least expressed gene of this pool, followed by TRPV4, TRPV1 and TRPV2. The comparison of TRPV1–4 gene expression between two groups of healthy and hyposensitive subjects highlighted the evident up-regulation of TRPV1, which was almost doubly expressed (1.9× normalized fold induction) in the latter group. All the four house-keeping genes tested in this work (Act-B, GAPDH, hCyPB, HPRT1) were classified as optimal controls and showed a constant expression in human leukocytes samples. We recommend the use of these genes in similar qRT-PCR studies on human blood cells.

## Background

Transient receptor potential (TRP) channels have been identified as cellular sensors that respond to diverse external and internal stimuli and play a fundamental role in the cytosolic free Ca^2+ ^concentration, either by acting as Ca^2+ ^entry pathways in the plasma membrane or via changes in membrane polarization [1].

The transient receptor potential vanilloid (TRPV) subfamily consists of the six mammalian members TRPV1–TRPV6 [2]. TRPV1–4 genes are related to thermal pain and/or warm sensing; in particular, TRPV1 is related to capsaicin, noxious temperature, pain perception, bladder distension and inflammation-induced thermal hyperalgesia [1,3]. Thermal sensation at high temperatures seems to be exclusively related to TRPV2 [4]. TRPV4 is also thought to play a fundamental role in nociception, osmosensing, warm sensing nociception and pressure sensing [2]. In the same vanilloid TRP gene subfamily, the TRPV5 and 6 genes are mainly involved in calcium re-absorption in the kidney and absorption in the duodenum [5].

In humans, disease-related changes in TRPV1 expression have been described in inflammatory bowel disease, irritable bowel syndrome, cervical cancer and destrusor hyperreflexia [6-9]. As a result, altered TRPV gene expression could play a role in the molecular diagnosis of various disease states. In particular, according to the proposed functions of TRPV1–4, these genes seem to play a fundamental role in breast and prostate cancer, myasthenic syndrome, non-insulin-dependent diabetes mellitus, central hypoventilation syndrome and cardiopathy (see [1] for a review). Abnormal regulation of ion channel function is especially interesting in all forms of inflammation and in systemic diseases [1].

For years, there was a general consensus that the expression of TRPV1 was restricted to sensory afferent neurones and discrete areas of the central nervous system. Later, however, the expression of this gene was also detected in a variety of non-neuronal tissues, including the bladder and urethral epithelium, bowel, lung, kidney, spleen, stomach, heart, oesophagus, neutrophil granulocytes, myocytes, and mast cells [10-13]. More recently, the expression of TRPV1 and 2 has also been detected in human peripheral blood cells (PBCs) [3]. The physiological role of TRPV1–2 genes in PBCs has yet to be determined, but it has been hypothesized that, under pathological conditions, their up-regulation may be an indicator of inflammation at a secondary site [3].

To study the roles of TRP channels in cells and tissues, it is important to know where and at what level they are expressed. For valid data on the specific expression in tissues, it is important to test gene expression by several independent techniques [14]. qRT-PCR is one of the most reliable and effective methods for the quantification of variations in mRNA expression, but the reliability of the results is strictly dependent on a careful experimental design, including the use of appropriate controls in the data normalization step [15-17]. Both absolute quantification and relative quantification procedures could lead to severe misinterpretation of the results if not preceded by preliminary evaluations of the house-keeping genes (HKGs) [17-20].

Unfortunately, our knowledge of the detailed mechanisms through which TRP channels function is still elementary and this situation hampers our understanding of the mechanistic role of these genes in human disease; the more we learn about the basic physiology of TRP channels and their potential role in disease, the closer we will come to the development of novel therapies for various disease states [2]. In this study, besides developing a Sybr Green qRT-PCR method based on a relative expression approach with accurately selected control genes, we quantify TRPV1–4 cDNA levels in human leukocytes by a comparison between 30 healthy subjects and a family with a peculiar form of hyposensitivity to pain, capsaicin and thermal stimuli.

## Results and discussion

### Real-Time amplifications

The primer pairs used to amplify the genes of interest (GOIs) (TRPV1–4) and the two HKGs (HPRT1 and hCyPB) were newly developed in this study and were accurately tested for their efficiency and reproducibility using serial 1:3 dilutions of the same template cDNA (Table 1). The oligonucleotides used to amplify the remaining two HKGs, Act-B and GAPDH, were taken from [21] and were checked before final acceptance on the basis of their reliability.

**Table 1:** Details of primers and amplicons for the 4 HKGs and the 4 GOIs analysed in this study.

Gene	Acc. Number	Forward Primer Sequence [5'→3']	Position in cDNA	Reverse Primer Sequence [5'→3']	Position in cDNA	Amplicon Length	E%	R^2^
Act-B*	NM_001101	CTGGAACGGTGAAGGTGACA	6^th^	AAGGGACTTCCTGTAACAATGCA	6^th^	140 bp	96.6	0.99
GAPDH*	NM_002046	TGCACCACCAACTGCTTAGC	7^th^	GGCATGGACTGTGGTCATGAG	7^th^/8^th^	87 bp	91.8	0.998
HPRT1	NM_000194	AGATGGTCAAGGTCGCAAG	6^th^	GTATTCATTATAGTCAAGGGCATATC	8^th^	128 bp	104.2	0.995
hCyPB	M60857	CCAACGCAGGCAAAGACACCAA	4^th^	GCTCTCCACCTTCCGCACCA	5^th^	131 bp	91.2	0.995
TRPV1	NM_080704	GGCTGTCTTCATCATCCTGCTGCT	12^th^	GTTCTTGCTCTCCTGTGCGATCTTGT	12^th^	117 bp	94.2	0.99
TRPV2	NM_016113	CTTCCTTTTCGGCTTCGCTGTAG	10^th^	GCACTGACTCTGTGGCATTGG	11^th^	95 bp	89.7	0.99
TRPV3	NM_145068	TCCTCACCTTTGTTCTCCTCCT	14^th^	CGCAAACACAGTCGGAAATCAT	15^th^	211 bp	103.6	0.995
TRPV4	NM_021625^† ^NM_147204^‡^	CTACGCTTCAGCCCTGGTCTC	11^th^/12^th†^	GCAGTTGGTCTGGTCCTCATTG	13^th ^^†^	76 bp	103.7	0.99

* refers to RT-PCR primers taken from [21]. † refers to TRPV4 isoform a and ‡ to TRPV4 isoform b.

All the primer pairs used in this study show good overall efficiency and excellent reproducibility of the amplification reactions (Table 1).

### Evaluation of the stability of the selected HKGs

To ensure correct normalization of the expression levels for the GOIs, the stability of the four control genes was statistically determined using the two VBA applets, *geNorm *and *NormFinder *[22] (Table 2). According to these two softwares, the four HKGs tested in this study show optimal overall reliability, confirmed by the extremely low "M" values (*geNorm*) and Stability Values (*NormFinder*) (Table 2). Therefore, all the HKGs were retained in the calculation of the normalization factor using REST2005 [23]. In particular, *geNorm *classifies GAPDH and Act-B as the best two controls of the group (Table 2), while the best position in the stability ranking produced by *NormFinder *is occupied by Act-B, followed by hCyPB, GAPDH and HPRT1 (Table 2). Moreover, *geNorm *suggests that an accurate normalization factor of qRT-PCR data can be calculated using only the two most stable HKGs (Act-B and GAPDH) with no further need to include additional controls in the analysis. As shown in Table 2, the addition of further HKGs will not significantly affect the reliability of the determined normalization factor, yielding V_2/3 _and V_3/4 _values (pair-wise variation between two sequential normalization factors) of 0.04 and 0.05 respectively, well below the default cut-off value of 0.15 [17]. The same analysis was repeated to evaluate the stability of the selected control genes in the group of pain insensitive subjects. Despite the poor statistical relevance of the results, due to the low number of samples included in this subanalysis, we obtained almost identical outcomes in the stability rankings of the selected controls, characterized by extremely low values of average expression stability for each gene.

**Table 2:** Stability ranking of the candidate reference genes calculated by geNorm and NormFinder and V values calculated by geNorm.

	Ranking				V value	
Software	1^st^	2^nd^	3^rd^	4^th^	V_2/3_	V_3/4_
***geNorm ***(Average M value)	**Act-B**/GAPDH* (0.013)		hCyPB (0.016)	**HPRT1 **(0.017)	0.005	0.004
***NormFinder ***(Average Stability Value)	**Act-B **(0.007)	hCyPB (0.008)	GAPDH (0.009)	**HPRT1 **(0.010)	Not determined	Not determined

* means that 1^st ^and 2^nd ^positions cannot be further ranked by *geNorm*.

### TRPV1–4 gene expression in leukocytes

**Figure 1:**
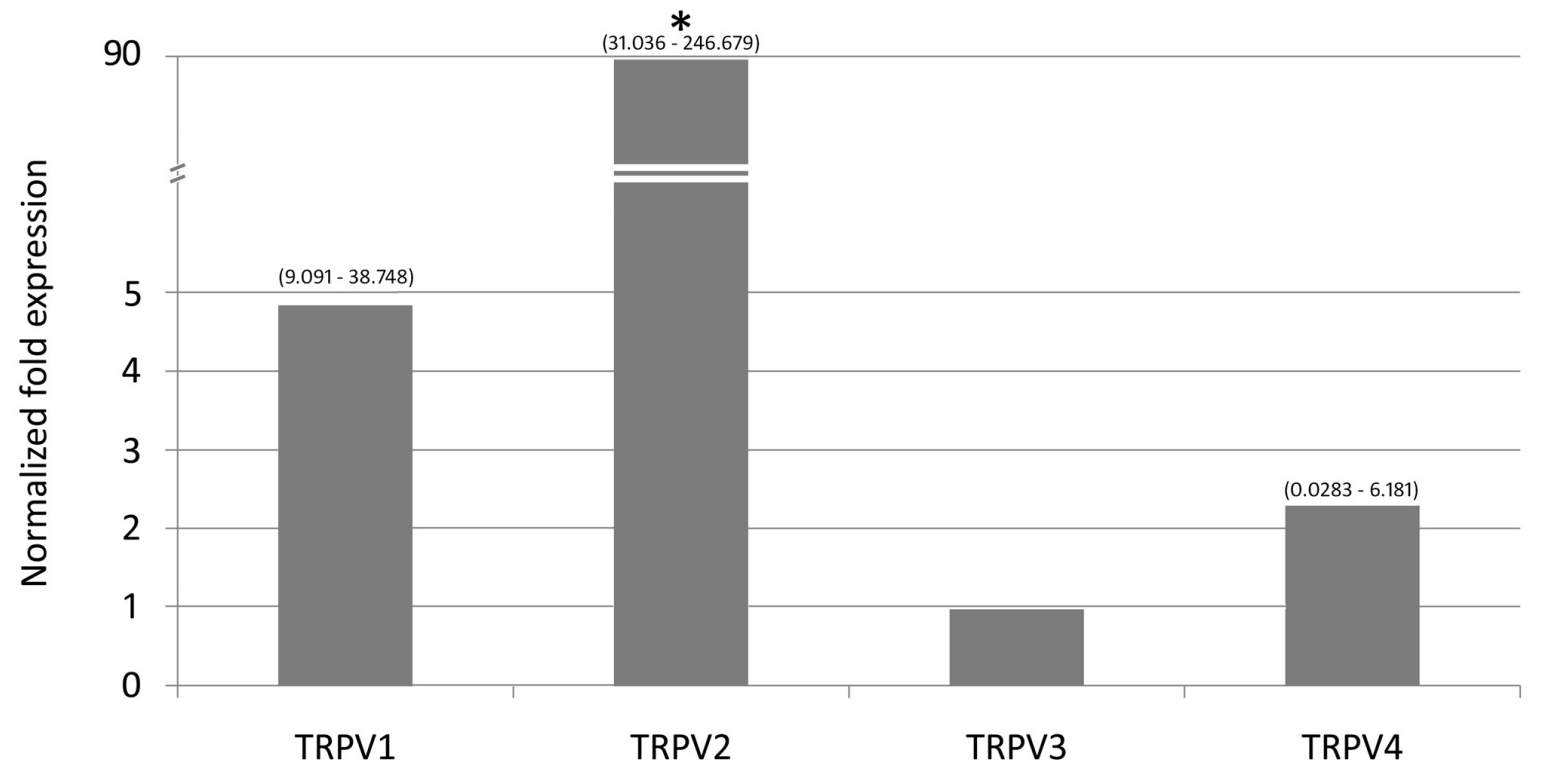
**Relative expression of the TRPV1–4 genes in human leukocytes from healthy subjects (n = 30)**. Relative expression measurements of the TRPV1–4 genes using qRT-PCR calculated using REST2005 (relative expression software tool). Fold change (y axis) represents the relative expression of the TRPV1, TRPV2 and TRPV4 genes in comparison to TRPV3 (least expressed gene of this pool and therefore considered equal to 1), normalized by Act-B, GAPDH, hCyPB and HPRT1 reference gene expressions. Expression standard errors are expressed within brackets at the top of each bar. * means p < 0.001.

**Figure 2:**
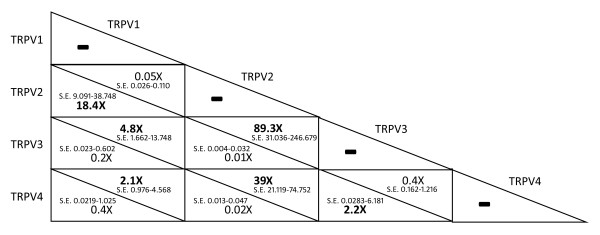
**Reciprocal expression levels of TRPV1–4 genes in human leukocytes from healthy donors (n = 30)**. Expression rates of TRPV1–4 genes calculated using qRT-PCR with relative quantification analysis using REST (relative expression software tool). Fold changes represents the reciprocal relative expression of the TRPV1–4 mRNA in physiological conditions in the 30 healthy donors. Values below (above) the diagonal line of each cell refer to the expression levels of genes on the y-axis (x-axis) compared to those of the corresponding genes on the x-axis (y-axis); S.E. means Expression Standard Error as calculated by the software REST; all p values are below 0.001.

The TRPV1–4 genes showed different expression patterns in leukocyte samples from the 30 healthy donors. TRPV3 was classified as the least expressed gene of the pool, followed by TRPV1 and TRPV4 (respectively 2.28 and 4.83 times more expressed than TRPV3). TRPV2 was by far the most expressed one (approximately 89.361 times more than TRPV3) (Fig. 1). The ratios of the TRPV1–4 gene expressions in the samples are summarized in Fig. 2. Each of the results presented in this section was confirmed by a *p *value < 0.001, calculated by REST2005. Despite the highly significant difference between mean expressions of the TRPV1–4 genes, our analysis is characterized by a broad variation of the standard errors. Considering that the blood samples analyzed in this study were checked for the amount of lymphocytes T and B by cytofluorimetric analysis, the broad variation in the standard error measurements could be due both to a different composition of samples between different cell subpopulations (neutrophils, eosinophils, basophils, monocytes, or macrophages), that could express at different levels the TRPV1–4 genes, or to variations in the expression levels in the same subpopulation.

### TRPV1–4 gene expression in healthy subjects

To evaluate possible sex differences, we compared the TRPV1–4 gene expression data for the men (n = 15) and women (n = 15) belonging to the control group of healthy subjects. None of the genes showed a significant difference in expression between males and females (TRPV1 *p = *0.1; TRPV2 *p *= 0.5; TRPV3 *p *= 0.7; TRPV4 *p *= 0.07).

Moreover, to evaluate the possible effects of hormonal changes during the menstrual cycle, we divided the 15 healthy females into two groups: those in the "follicular phase" (8 subjects) and those in the "luteal phase" (7 subjects). Also in this case, the TRPV1–4 expression did not differ significantly between the two groups (TRPV1 *p *= 0.9; TRPV2 *p *= 0.6; TRPV3 *p *= 0.4 TRPV4 *p *= 0.3).

### TRPV1–4 gene expression differences between healthy and hyposensitive subjects

Interestingly, the statistical analysis of the TRPV1–4 gene expression differences between healthy donors (n = 30) and subjects hyposensitive to painful thermal stimuli and capsaicin stimulation (n = 5) showed that TRPV1 gene expression was almost doubled in the hyposensitive patients (mean induction factor of TRPV1 is 1.9; *p = *0.023; Standard Error range 1.017–3.540; Table 3). However, the TRPV2–4 gene expression did not differ significantly between the two groups (Table 3).

**Table 3:** TRPV1–4 relative expression measure comparisons between the group of subjects affected by hyposensitivity to capsaicin, pain and thermal stimuli and the group of healthy controls.

**Gene**	Normalized Fold Expression	Expression Standard Error	P value
**TRPV1**	1.9	1.017 – 3.540	0.023*
**TRPV2**	0.9	0.592 – 1.474	0.941
**TRPV3**	1.6	0.391 – 6.803	0.23
**TRPV4**	1.5	0.746 – 3.084	0.123

Data are given as normalized fold expression, indicating the relative quantification of the qRT-PCR signal detected in hyposensitive subjects (n = 5) in relation to that detected in healthy donors (n = 30). Note that the significance (*) for the TRPV1 gene indicates an up-regulation in subjects affected by hyposensitivity to capsaicin, pain and thermal stimuli.

## Conclusion

In the present study, we demonstrated that the TRPV1–4 genes are expressed in human leukocytes. While the rates of expression were not influenced by gender, we found significant differences for TRPV1 between healthy subjects and patients hyposensitive to painful thermal and capsaicin stimulation.

The genes belonging to the transient receptor potential (TRP) family, and in particular the members of the TRPV subfamily, are known to be involved in cell responses to internal and external environmental factors, including osmolarity, pressure, heat, cold, hormones and inflammatory mediators [11]. Blood is of particular interest among the non-nervous biological material because of its involvement in immune and inflammatory processes and its role in the distribution of circulating signals (cytokines, hormones). A recent study demonstrating TRPV1–2 gene expression in human peripheral blood cells by means of qRT-PCR and immunocytochemical techniques [3], stressed the importance of further studies to clarify the physiological and pathophysiological role and regulation of TRPV channels in human mononuclear cells. In this context, our work constitutes an additional effort to investigate the presence and relative amounts of TRPV1–4 gene transcripts in human leukocytes.

For this purpose, we decided to test and validate our Sybr Green qRT-PCR assay based on the relative quantification of TRPV1–4 gene transcripts. While absolute quantification (based on external standards) is the method of choice for nucleic acid quantification, relative quantification is based on the expression levels of one or more target genes versus one or more reference genes and, in many experiments, is adequate for investigating physiological changes in gene expression levels [17,24]. The normalization procedure should be considered mandatory in qRT-PCR studies and the importance of the choice of the most stably expressed HKGs is too often underestimated during qRT-PCR assays, easily leading to misinterpretation and low reproducibility of the final results. Therefore, we decided to validate, up to their expression stability, four of the most widely used house-keeping genes in human leukocyte samples from the 30 healthy donors, equally divided between males and females. Amplicon lengths and specificity, and PCR amplification efficiencies and reproducibility, were accurately checked, and the stability of the selected control gene expressions among samples was analysed with two widely used softwares, namely *geNorm *and *NormFinder*. All four house-keeping genes considered in this study (Act-B, GAPDH, hCyPB, HPRT1) were classified as optimal controls and showed stable expression in human leukocytes samples. We recommend the use of these genes for similar qRT-PCR studies and we hope that our outcomes will help to draw up a preliminary "universal protocol" for a better comparison of qRT-PCR results from different studies on human blood cells.

In addition to proving the presence of TRPV1–4 cDNAs in human leukocytes, our qRT-PCR assay revealed a lack of significant differences in TRPV1–4 expression between healthy males (n = 15) and females (n = 15), and between females sampled during the luteal and follicular phases of the menstrual cycle. This partially contrasts with experimental data concerning the effect of estrogens on the expression of the TRPV genes. For instance, the TRPV1 expression in viscera was found to be increased by estradiol administration in female rats [25], a pattern that seems not to be confirmed in our experiments on human leukocyte samples. Therefore, the physiological hormonal differences between women in the two menstrual phases, as well as the hormonal differences between the sexes, failed to affect TRPV1–4 gene expression, suggesting a negligible influence of gonadal hormones on the haematic expression of the TRPV subfamily genes, at least in healthy human subjects.

Additionally, the qRT-PCR assays allowed us to determine the relative expression of TRPV1–4 genes in human leukocytes. While the presence of TRPV1–2 transcripts in human blood cells was recently reported, our study is the first to demonstrate TRPV3–4 expression in human leukocytes. According to the results for the 30 healthy donors, we can conclude that TRPV3 is the least expressed gene of this pool, followed by TRPV4, TRPV1 and TRPV2 (Fig. 1). Compared to TRPV3, the TRPV4, TRPV1 and TRPV2 genes showed a physiological normalized fold expression of 2.2×, 4.8× and 89.3×, respectively. The outcomes of our analysis (Fig. 2) are in partial agreement with the data reported by [3], where a ≈150-fold over-expression of TRPV2 compared to TRPV1 is shown. Although substantially reducing the gap between the expression of the two genes, our results confirm this trend: the TRPV1 cDNA levels are 18.4-fold lower than those of TRPV2.

The comparison of TRPV1–4 gene expression between healthy subjects (n = 30) and the hyposensitive group (n = 5) highlighted the evident up-regulation of TRPV1, which was almost doubly expressed (1.9× normalized fold induction) in the latter group (Table 3). TRPV1 is by far the best understood TRP channel [1] and both in vivo and in vitro studies have repeatedly shown that these receptors (TRPV1–4) are involved in the transmission of pain and/or thermal stimuli and capsaicin sensation [26,27]. In particular, TPRV1 gene transcription produces an mRNA coding for a protein devoted to the vanilloid receptor 1; it is recognized as a molecular integrator of inflammatory mediators and is thought to mediate peripheral sensitization, involving a reduced threshold of activation and an increased responsiveness of peripheral nociceptors [28-31].

On the basis of these findings, the higher TRPV1 expression, related to the influence of TRPV genes in the immune response of lymphocytes, might be caused by the more frequent inflammatory and infectious processes in these patients due to their hyposensitive status. Under pathological conditions, the up-regulation of TRPV1 could be an indicator of inflammation at a secondary site related to the influence of TRPV1 on the release of neuropeptides that can up-regulate the expression of adhesion molecules in endothelial cells and consequently activate T-cells [3].

The up-regulation of TRPV1 in pain-insensitive subjects may also be linked to its polymorphism [32,33] or to some functional anomalies of this gene [34] that may be associated with pain perception.

Two recent publications focused on the functional effects of nonsynonymous polymorphism in the human TRPV1 gene and on the genetic influence on variability in human pain sensitivity [32,33]. TRPV1 presents five nonsynonymous polymorphisms expected to result in nonconservative amino acid substituitions [33]. While TRPV1^WT ^and its variant forms show similar EC_50 _for capsaicin, HEK293 cells transfected with TRPV1^P91S ^and TRPV1^I315M ^variants exhibit enhanced responsiveness to a second TRPV1 agonist, named anandamide [33]. An additional sixth single nucleotide polymorphism, predicted to reside within membrane-spanning helix five, seems to be responsible for a conservative amino acid substitution (TRPV1^I585V^) that could alter receptor structure/function during cold perception [32], increasing sensitivity to cold-induced pain in American females subjects. This observation is apparently in contrast with the previously reported in vitro normal functional response to agonists of the TRPV1^I585V ^variant [33,35]. In the heterologous expression model described in [33], the two allelic variants TRPV1^I315M ^and TRPV1^P91S ^resulted in markedly increased abundance of the variant TRPV1 protein, although with a very modest increment in the mRNA levels; the authors suggest that disease state is mediated through altered expression of a normal protein and that much of the intersubject phenotypic variation will be encoded by polymorphisms that influence levels of gene expression.

A TRPV1 splice variant lacking Exon 7 (named TRPV1b) was previously cloned from human dorsal root ganglia [34], showing that recombinant TRPV1b was not activated by capsaicin (1 microM), protons (pH 5.0) or heat (50 degrees C). When co-expressed with TRPV1, TRPV1b formed complexes with TRPV1, and dose-dependently inhibited TRPV1 channel function in response to capsaicin, acidic pH, heat and endogenous vanilloids. These data support the hypothesis that TRPV1b is a naturally existing inhibitory modulator of TRPV1 and that mutual regulation between TRPV1 and TRPV1b might take place in the same neuronal cell *in vivo*. Therefore, in hyposensitive subjects, the up-regulation of TRPV1b could lead to pathological inhibition of TRPV1 activity, with a consequent loss of the natural ability to perceive thermal, pain and capsaicin stimulation. Other authors [34] did not exclude the possibility that TRPV1b may be up-regulated under physiological or pathological conditions. Since the qRT-PCR method described in this study does not allow discrimination between cDNAs transcribed from the TRPV1 gene or the recombinant TRPV1b splice variant, further studies are required to clarify the potential role of the TRPV1b splice variant and to quantify its expression in tissues of hyposensitive subjects.

It is evident that the presence of the TRPV1 splice variant or of genetic polymorphism associated with this gene, may provide another mechanism for down-regulating channel activity, and the more we learn about fundamental TRP channel physiology and the potential role of TRPs in disease, the closer we will come to the development of novel therapies for various disease states.

## Methods

### Healthy subjects

The control group of 30 healthy volunteers consisted of 15 men (mean age 35.0, range 25–45 years) and 15 women (mean age 33.3, range 22–46 years). Subjects were asked to fill in a short questionnaire regarding their general state of health and, in particular, their sensitivity to painful thermal stimuli and capsaicin perception. For the women, the menstrual cycle phase was recorded and considered in the analysis as follicular (1^st^–14^th ^day) or luteal (15^th^–28^th ^day). Written informed consent was obtained from all subjects.

### Hyposensitive subjects

This sample consisted of five subjects previously diagnosed as suffering from hyposensitivity to painful thermal and capsaicin stimulation. The subjects, belonging to the same family (3 women and 2 boys from 3 generations), were a 75-year-old woman, her two daughters (40-year-old and 34-year-old sisters) and two nephews (two brothers, 14 and 11 years old). All these subjects were apparently healthy, with normal mental performance and high performance in physical activities, with an apparently normal quality of life. The main clinical data were: absence of pain in response to usual painful stimuli (all subjects), low sensitivity to capsaicin (all subjects), history of clinical autonomic disability plus several episodes of hyperthermia, increased warm and cold thresholds, recurrent infections (3 subjects), absence of corneal reflex (all subjects), rare episodes of orthostatic hypotension (3 subjects). Written informed consent was obtained from the adults and from the parents of the two children; oral consent was also obtained from the participating children.

### RNA isolation

Peripheral blood (3 ml) was collected in sterile tubes from the 30 healthy donors (15 males, 15 females) and the five patients affected by PTCH. Cytofluorimetric analysis of samples revealed a marked uniformity of lymphocytes T and B populations. The markers CD3 and CD19 were respectively used to detect lymphocytes T and B. Heparin was used to stabilize the samples and cellular RNA was immediately isolated from whole blood (1.5 ml) using the QIAamp RNA Blood Mini Kit (Qiagen). A DNase on-column treatment was performed during the RNA isolation procedure to avoid genomic DNA (gDNA) carryovers (RNase-Free DNase Set, Qiagen). The concentration and purity of all RNA samples, eluted in a final volume of 35 μl RNase-Free water (Qiagen), were determined with a Nano-Drop^® ^ND-1000 UV-Vis Spectrophotometer (NanoDrop Technologies). Based on the absorbance ratio at 260/280 nm and at 230/260 nm, all samples were pure and free of protein and organic pollutants derived from the RNA isolation procedure. The overall sample integrity was confirmed by denaturing formaldehyde agarose gel (1.2%) electrophoresis and ethidium-bromide staining, showing sharp and intense 18S and 28S ribosomal RNA bands with a total absence of smears. Samples were stored at -80°C until use.

### Reverse transcription

To avoid any genomic DNA contamination during qRT-PCR, retrotranscription of 600 ng total RNA was performed with the QuantiTect Reverse Transcription Kit (Qiagen), according to the manufacturer's instructions. This kit ensures complete digestion of genomic DNA by a brief incubation of the sample at 42°C with a specific Wipeout buffer before retrotranscription. For each of the 30 samples, 1 μl of total RNA was collected prior to the retrotranscription step and stored at -80°C. RNA samples were subsequently used in Real-Time PCR, as internal controls to check for potential gDNA contamination.

### Design of RT-PCR primers

Real-Time PCR primers used to amplify the two control genes, β-actin and GAPDH, were taken from [21].

Nucleotide sequences of the four genes of interest (GOIs) and the remaining two house-keeping genes (HKGs; HPRT1 and hCyPB) were downloaded from GenBank files under the Accession Numbers specified in Table 1 and specific exon-intron boundaries were identified. PCR primers for real-time assays were designed using Beacon Designer 2.06 (Premier Biosoft International) giving special attention to primer length, annealing temperature, base composition and 3'-end stability. To ensure optimal DNA polymerization efficiency, the amplicon length ranged between 76 and 211 bp (Table 1). All the RT-PCR primer pairs used in this study span exon-exon junctions or are located on different exons, with the exceptions of those for the TRPV1 and Act-B genes (Table 1). TRPV4 primers were specifically designed to amplify a short amplicon located in proximity to the 3' end of the cDNA, shared between the two described splice variants, isoform a (GenBank accession number NM_021625) and isoform b (NM_147204). During preliminary qRT-PCR assays, the optimal primer concentration was determined for each primer pair: it generated the lowest Ct value and a sharp peak, with no amplification of non-specific products or primer-dimer artifacts. For each pair of primers, the efficiency of qRT-PCR (E), the slope values and the correlation coefficients (R2) were determined, using serial 1:3 dilutions of template cDNA, on a iQ5 machine (Bio-Rad) (Table 1). Products were subsequently run on 2% agarose gel to check for size specificity and eventually sequenced.

### Real-Time PCR

Real-time amplifications, using SYBR Green detection chemistry, were run in triplicate on 96-well reaction plates with the iQ5 machine (Bio-Rad). Reactions were prepared in a total volume of 20 μl containing: 0.8 μl cDNA, 0.6 μl of each 10 μM primer (300 mM each; Invitrogen), 10 μl of iQ™ SYBR^® ^Green Supermix (Bio-Rad) and 8 μl RNase/DNase-free sterile water (Qiagen). Blank controls were run in triplicate for each master mix. The cycle conditions were set as follows: initial template denaturation at 95°C for 1 min, followed by 40 cycles of denaturation at 95°C for 10 s, and combined primer annealing/elongation at 60°C for 30 s, as described in [36]. This cycle was followed by a melting curve analysis, ranging from 56°C to 95°C, with temperature increasing by steps of 0.5°C every 10 s.

Baseline and threshold values were automatically determined for all plates using the Bio-Rad iQ5 Software 1.0. In order to ensure comparability between data obtained from different experimental plates, the threshold value was subsequently manually set to the value corresponding to the arithmetic mean between the automatically determined thresholds annotated previously; then all data were reanalyzed. Raw Ct values (See additional file 1: Raw Ct values table) were transformed to quantities using an Excel spreadsheet generated by the authors, based on the comparative Ct method. The resulting data were converted into correct input files, according to the requirements of the software, and analysed using the Excel spreadsheet REST2005 [23] and the VBA applet *geNorm *[17].

### Reliability of the HKGs

To ensure correct normalization of the expression levels for the GOIs, the stability of the four control genes was statistically determined using the two VBA applets *geNorm *[17] and *NormFinder *[22] (Table 2). *geNorm *provides a ranking of the tested genes based on their expression stability, determining the two most stable HKGs or a combination of multiple stable genes for normalization. Selected HKGs are ranked according to the determined control gene-stability measure (M, average pair-wise variation of a particular gene with all other control genes), from the most stable (lowest M values) to the least stable (highest M values) [17,37]. In addition, assessment of the normalization factor allows identification of the optimal number of control genes.

*NormFinder *is another VBA applet based on an algorithm for identifying the optimal normalization gene(s) among a set of candidates. It ranks the set of candidate genes according to their expression stability value in a given sample set and a given experimental design [22].

### REST2005 analysis

REST2005 is a software tool based on qRT-PCR raw data which allows a relative quantification between two sample groups and subsequently tests the significance of the results with a suitable statistical model [23]. This software determines whether there is a significant difference between two groups of samples, taking into account issues of reaction efficiency and reference gene(s) normalization and using randomisation techniques. In this study, REST2005 was specifically used to compare the expression levels of the TRPV1–4 genes in whole blood samples (normalized using the 4 HKGs previously analyzed by *geNorm *and *NormFinder*) of various groups: (i) two groups of healthy subjects consisting of 15 males and 15 females; (ii) two groups of females in the luteinic (*n *= 7) and follicular (*n *= 8) phase of the menstrual cycle; (iii) healthy (n = 30) and hyposensitive (*n *= 5) subjects. The same software was also used to calculate relative expression levels of the TRPV1–4 genes for all the healthy donors (*n *= 30).

Statistical analysis of gene expression was based on an hypothesis test performing 50,000 random reallocations of samples (hyposensitive subjects) and controls (healthy subjects). The mathematical model used was based on the correction for exact PCR efficiencies and the mean crossing point deviation between sample group(s) and control group(s). Subsequently, the expression ratio results of the investigated transcripts were tested for significance by a Pair Wise Fixed Reallocation Randomisation Test and plotted using standard error (SE) estimation via a complex Taylor algorithm [23]. Samples with a probability value of < 0.05 were considered significantly different between the groups.

## Competing interests

The authors declare that they have no competing interests.

## Authors' contributions

GS performed all the experimental procedure and was primary author of the manuscript. CP participated in the experimental process and data analysis. AMA and LM provided the samples. AMA, RZ, IC, FF and VB supervised the study design and contributed to writing the manuscript.

## Supplementary Material

Additional file 1 **Raw Ct values table.** The table shows all the Ct values collected in this study both for the house-keeping genes (Act-B, Gapdh, Hprt1, and hCyPB) and the genes of interest (TRPV 1–4) in the 30 healthy individuals and in the 5 hyposensitive patients. The ymbol ♀ refers to females and the symbol ♂ refers to; both the 2 symbols are followed by a progressive number linked to the corresponding individual. The symbol * refers to hyposensitive subjects. Click here for file

## References

[B1] Nilius B (2007). TRP channels in disease. Biochim Biophys Acta.

[B2] Nilius B, Owsianik G, Voets T, Peters JA (2007). Transient receptor potential cation channels in disease. Physiol Rev.

[B3] Saunders CI, Kunde DA, Crawford A, Geraghty DP (2007). Expression of transient receptor potential vanilloid 1 (TRPV1) and 2 (TRPV2) in human peripheral blood. Mol Immunol.

[B4] Benham CD, Davis JB, Randall AD (2002). Vanilloid and TRP channels: a family of lipid-gated cation channels. Neuropharmacology.

[B5] Nijenhuis T, Hoenderop JG, Bindels RJ (2005). TRPV5 and TRPV6 in Ca(2+) (re)absorption: regulating Ca(2+) entry at the gate. Pflugers Arch.

[B6] Yiangou Y, Facer P, Dyer NH, Chan CL, Knowles C, Williams NS, Anand P (2001). Vanilloid receptor 1 immunoreactivity in inflamed human bowel. Lancet.

[B7] Tympanidis P, Casula MA, Yiangou Y, Terenghi G, Dowd P, Anand P (2004). Increased vanilloid receptor VR1 innervation in vulvodynia. Eur J Pain.

[B8] Tominaga M, Caterina MJ (2004). Thermosensation and pain. J Neurobiol.

[B9] Planells-Cases R, Garcia-Sanz N, Morenilla-Palao C, Ferrer-Montiel A (2005). Functional aspects and mechanisms of TRPV1 involvement in neurogenic inflammation that leads to thermal hyperalgesia. Pflugers Arch.

[B10] Birder LA, Kanai AJ, de Groat WC, Kiss S, Nealen ML, Burke NE, Dineley KE, Watkins S, Reynolds IJ, Caterina MJ (2001). Vanilloid receptor expression suggests a sensory role for urinary bladder epithelial cells. Proc Natl Acad Sci USA.

[B11] Gunthorpe MJ, Benham CD, Randall A, Davis JB (2002). The diversity in the vanilloid (TRPV) receptor family of ion channels. Trends Pharmacol Sci.

[B12] Heiner I, Eisfeld J, Luckhoff A (2003). Role and regulation of TRP channels in neutrophil granulocytes. Cell Calcium.

[B13] Stokes AJ, Wakano C, Del Carmen KA, Koblan-Huberson M, Turner H (2005). Formation of a physiological complex between TRPV2 and RGA protein promotes cell surface expression of TRPV2. J Cell Biochem.

[B14] Kunert-Keil C, Bisping F, Kruger J, Brinkmeier H (2006). Tissue-specific expression of TRP channel genes in the mouse and its variation in three different mouse strains. BMC Genomics.

[B15] Hendriks-Balk MC, Michel MC, Alewijnse AE (2007). Pitfalls in the normalization of real-time polymerase chain reaction data. Basic Res Cardiol.

[B16] Huggett J, Dheda K, Bustin S, Zumla A (2005). Real-time RT-PCR normalisation; strategies and considerations. Genes Immun.

[B17] Vandesompele J, De Preter K, Pattyn F, Poppe B, van Roy N, De Paepe A, Speleman F (2002). Accurate normalization of real-time quantitative RT-PCR data by geometric averaging of multiple internal control genes. Genome Biol.

[B18] Bemeur C, Ste-Marie L, Desjardins P, Hazell AS, Vachon L, Butterworth R, Montgomery J (2004). Decreased beta-actin mRNA expression in hyperglycemic focal cerebral ischemia in the rat. Neurosci Lett.

[B19] Pfaffl MW, Tichopad A, Prgomet C, Neuvians TP (2004). Determination of stable housekeeping genes, differentially regulated target genes and sample integrity: BestKeeper – Excel-based tool using pair-wise correlations. Biotechnol Lett.

[B20] Selvey S, Thompson EW, Matthaei K, Lea RA, Irving MG, Griffiths LR (2001). Beta-actin – an unsuitable internal control for RT-PCR. Mol Cell Probes.

[B21] Vandesompele J, De Preter K, Pattyn F, Poppe B, Van Roy N, De Paepe A, Speleman F (2002). Accurate normalization of real-time quantitative RT-PCR data by geometric averaging of multiple internal control genes. Genome Biol.

[B22] Andersen CL, Jensen JL, Orntoft TF (2004). Normalization of real-time quantitative reverse transcription-PCR data: a model-based variance estimation approach to identify genes suited for normalization, applied to bladder and colon cancer data sets. Cancer Res.

[B23] Pfaffl MW, Horgan GW, Dempfle L (2002). Relative expression software tool (REST) for group-wise comparison and statistical analysis of relative expression results in real-time PCR. Nucleic Acids Res.

[B24] Pfaffl MW (2001). A new mathematical model for relative quantification in real-time RT-PCR. Nucleic Acids Res.

[B25] Yan T, Liu B, Du D, Eisenach JC, Tong C (2007). Estrogen amplifies pain responses to uterine cervical distension in rats by altering transient receptor potential-1 function. Anesth Analg.

[B26] Levine JD, Alessandri-Haber N (2007). TRP channels: targets for the relief of pain. Biochim Biophys Acta.

[B27] Venkatachalam K, Montell C (2007). TRP channels. Annu Rev Biochem.

[B28] Barton NJ, McQueen DS, Thomson D, Gauldie SD, Wilson AW, Salter DM, Chessell IP (2006). Attenuation of experimental arthritis in TRPV1R knockout mice. Exp Mol Pathol.

[B29] Caterina MJ, Leffler A, Malmberg AB, Martin WJ, Trafton J, Petersen-Zeitz KR, Koltzenburg M, Basbaum AI, Julius D (2000). Impaired nociception and pain sensation in mice lacking the capsaicin receptor. Science.

[B30] Szallasi A, Blumberg PM (1999). Vanilloid (Capsaicin) receptors and mechanisms. Pharmacol Rev.

[B31] Lotsch J, Geisslinger G (2007). Current evidence for a modulation of nociception by human genetic polymorphisms. Pain.

[B32] Kim H, Neubert JK, San Miguel A, Xu K, Krishnaraju RK, Iadarola MJ, Goldman D, Dionne RA (2004). Genetic influence on variability in human acute experimental pain sensitivity associated with gender, ethnicity and psychological temperament. Pain.

[B33] Xu H, Tian W, Fu Y, Oyama TT, Anderson S, Cohen DM (2007). Functional effects of nonsynonymous polymorphisms in the human TRPV1 gene. Am J Physiol Renal Physiol.

[B34] Vos MH, Neelands TR, McDonald HA, Choi W, Kroeger PE, Puttfarcken PS, Faltynek CR, Moreland RB, Han P (2006). TRPV1b overexpression negatively regulates TRPV1 responsiveness to capsaicin, heat and low pH in HEK293 cells. J Neurochem.

[B35] Hayes P, Meadows HJ, Gunthorpe MJ, Harries MH, Duckworth DM, Cairns W, Harrison DC, Clarke CE, Ellington K, Prinjha RK (2000). Cloning and functional expression of a human orthologue of rat vanilloid receptor-1. Pain.

[B36] Radonic A, Thulke S, Mackay IM, Landt O, Siegert W, Nitsche A (2004). Guideline to reference gene selection for quantitative real-time PCR. Biochem Biophys Res Commun.

[B37] Spinsanti G, Panti C, Lazzeri E, Marsili L, Casini S, Frati F, Fossi CM (2006). Selection of reference genes for quantitative RT-PCR studies in striped dolphin (Stenella coeruleoalba) skin biopsies. BMC Mol Biol.

