# Supplementary material for: Quantitative Real-Time PCR detection of TRPV1–4 gene expression in human leukocytes from healthy and hyposensitive subjects
Source: Mol Pain. 2008 Nov 4;4:51. doi: 10.1186/1744-8069-4-51 (PMC2588574; doi:10.1186/1744-8069-4-51)
Supplement: Additional file 1 — Raw Ct values table. The table shows all the Ct values collected in this study both for the house-keeping genes (Act-B, Gapdh, Hprt1, and hCyPB) and the genes of interest (TRPV 1–4) in the 30 healthy individuals and in the 5 hyposensitive patients. The ymbol ♀ refers to females and the symbol ♂ refers to; both the 2 symbols are followed by a progressive number linked to the corresponding individual. The symbol * refers to hyposensitive subjects. [file 1744-8069-4-51-S1.pdf]

	House-keeping Genes				Genes of interest			
**Samples**	**Act-B**	**Gapdh**	**Hprt1**	**hCyPB**	**TRPV1**	**TRPV2**	**TRPV3**	**TRPV4**
♀**1**	14.75	18.5	23.05	20.71	28.82	24.41	30.87	30.2
♀**2**	15.02	19.04	23.83	21.3	29.02	25.83	30.79	30.89
♀**3**	15.7	19.31	23.85	21.93	29.18	25.72	31.86	29.76
♀**4**	14.71	18.77	23.21	21.02	28.63	24.68	32.44	30.77
♀**5**	14.95	18.83	23.31	21.23	29.05	24.99	32.5	29.65
♀**6**	14.77	18.35	22.85	21.24	29.44	25.33	31.66	31
♀**7**	15.81	19.57	24.29	21.92	30.33	26.61	33.4	32.56
♀**8**	14.7	18.95	23.43	21.01	30.35	25	31.92	30.52
♀**9**	14.94	19	23.64	21.55	29.93	25.51	32.89	30.14
♀**10**	14.84	18.82	23.55	20.83	29.92	25.03	33.56	30.05
♀**11**	15	19	23.64	21.43	30.04	24.96	31.11	30.62
♀**12**	14.66	18.64	23.35	21.08	30	24.58	32.59	30.75
♀**13**	15.65	19.24	24.01	21.71	30.29	26.88	34.12	31.2
♀**14**	15.01	18.96	23.8	21.32	29.01	25.53	30.78	30.82
♀**15**	14.91	19.02	23.7	21.55	29.88	25.44	32.85	30.08
♂**1**	15.15	18.99	23.6	21.16	31.33	24.88	33.52	31.97
♂**2**	14.42	18.27	23.25	20.78	30.96	25.27	33.77	29.73
♂**3**	14.63	18.66	23.36	21.02	29.4	24.85	31.71	30.61
♂**4**	14.94	19.26	23.49	21.31	31.16	25.13	32.14	32.87
♂**5**	14.81	18.85	23.26	21.01	30.33	24.75	31.77	31.41
♂**6**	14.6	18.84	23.05	20.93	28.22	24.76	30.42	30.44
♂**7**	15.2	19.25	23.72	21.23	29.18	25.66	32.87	30.83
♂**8**	14.63	18.63	23.15	20.79	29.15	24.78	29.72	31.09

♂**9**	14.88	19.02	24.26	21.71	30.02	25.87	33.83	31.38
♂**10**	14.87	18.75	23.51	21.3	30.51	25.68	33.47	31.59
♂**11**	14.5	18.52	22.99	20.75	27.8	28.49	30.04	30.5
♂**12**	14.94	19.05	23.34	21.13	32.29	25.69	32.78	31.16
♂**13**	14.62	18.25	23.53	21.11	29.65	24.92	33.09	30.16
♂**14**	14.85	18.74	23.17	21.27	29.46	24.7	31.52	31.61
♂**15**	15.26	19.11	23.89	21.65	29.13	25.25	29.97	31.38
*****♀**1**	15.01	19.27	23.95	21.51	28.75	25.12	30.6	30.97
*****♀**2**	15.23	19.42	23.84	21.59	29.27	25.77	33.79	30.18
*****♀**3**	15.85	19.31	23.98	22	29.81	26.5	33.41	31.67
*****♂**1**	15.03	18.73	23.87	20.99	28.88	25.89	30.65	29.87
*****♂**2**	15.69	19.3	23.77	21.16	28.96	25.63	30.92	30.06
Minimum Ct	14.42	18.25	22.85	20.71	27.8	24.41	29.72	29.65
Maximum Ct	15.85	19.57	24.29	22	32.29	28.49	34.12	32.87
